# The orbitofrontal cortex of the sheep. Topography, organization, neurochemistry, digital tensor imaging and comparison with the chimpanzee and human

**DOI:** 10.1007/s00429-022-02479-w

**Published:** 2022-03-26

**Authors:** Tommaso Gerussi, Jean-Marie Graïc, Annamaria Grandis, Antonella Peruffo, Bruno Cozzi

**Affiliations:** 1 Department of Comparative Biomedicine and Food Science (BCA), University of Padova, Viale dell’Università, 16, 35020 Legnaro, PD Italy; 2 Department of Veterinary Medical Sciences, University of Bologna, Via Tolara di Sopra, 43, 40064 Ozzano dell’Emilia, BO Italy

**Keywords:** Orbitofrontal cortex, DTI, Sheep, Human, Chimpanzee

## Abstract

Areas dedicated to higher brain functions such as the orbitofrontal cortex (OFC) are thought to be unique to hominidae. The OFC is involved in social behavior, reward and punishment encoding and emotional control. Here, we focused on the putative corresponding area in the sheep to assess its homology to the OFC in humans. We used classical histology in five sheep (*Ovis aries*) and four chimpanzees (*Pan troglodytes*) as a six-layered-cortex primate, and Diffusion Tensor Imaging (DTI) in three sheep and five human brains. Nissl’s staining exhibited a certain alteration in cortical lamination since no layer IV was found in the sheep. A reduction of the total cortical thickness was also evident together with a reduction of the prevalence of layer one and an increased layer two on the total thickness. Tractography of the sheep OFC, on the other hand, revealed similarities both with human tracts and those described in the literature, as well as a higher number of cortico-cortical fibers connecting the OFC with the visual areas in the right hemisphere. Our results evidenced the presence of the basic components necessary for complex abstract thought in the sheep and a pronounced laterality, often associated with greater efficiency of a certain function, suggested an evolutionary adaptation of this prey species.

## Introduction

The orbitofrontal cortex of primates (OFC) represents the ventral part of the frontal lobe that receives projections from the medial (magnocellular) nucleus of the mediodorsal thalamus (MDmc) (Fuster 1997, 2015; Kringelbach and Rolls 2004). Phylogenetically, it belongs to one of the most recent neocortical area, the prefrontal cortex (PFC), so much so that in primates it is thought to have “displaced” the other areas caudally (Van Eden and Uylings 1985). The OFC is involved in integration of multi-sensory inputs, reward and punishment encoding in addition to emotional regulation (Hof et al. 1995).

Inputs to the OFC derive from all sensory areas, including visceral inputs but also from the amygdala, the anterior and posterior cingulate cortices, motor cingulate area, premotor area F5, other prefrontal areas and the hippocampus (Öngür and Price 2000; Kringelbach and Rolls 2004). Outputs mostly correspond to inputs with some differences such as strong connections with the posterior hypothalamus and the periaqueductal gray (Kringelbach and Rolls 2004).

The topography of the (human) OFC includes Brodmann areas 10, 11 and 47 (Brodmann 1909). Due to its complexity, subsequent studies further subdivided the human OFC into more areas and subareas (10, 11, 47/12, 13 and 14), thus partially solving discrepancies with the macaque (Walker 1940; Carmichael et al. 1994; Petrides and Pandya 1994). For the present study, we considered the human OFC as the region that receives the major inputs from the MDmc (Guigere and Goldman-Rakic 1988; Ray and Price 1993), thus including Walker’s (1940) and Petrides and Pandya’s (1994) areas 11, 47/12, 13 and 14. These areas comprise the orbital part of the inferior frontal gyrus, the gyrus rectus, the olfactory sulcus, lateral orbital sulci and gyri notwithstanding individual sulcal variability (Ono et al. 1990; Kringelbach and Rolls 2004; Petrides and Pandya 2012). The cytoarchitectonics of the human OFC is characterized by well-developed layers V and VI, and by a relevant layer IV, the thickness of which increases in a caudo-rostral direction (Hof et al. 1995; Petrides and Pandya 2012).

**Fig. 1:**
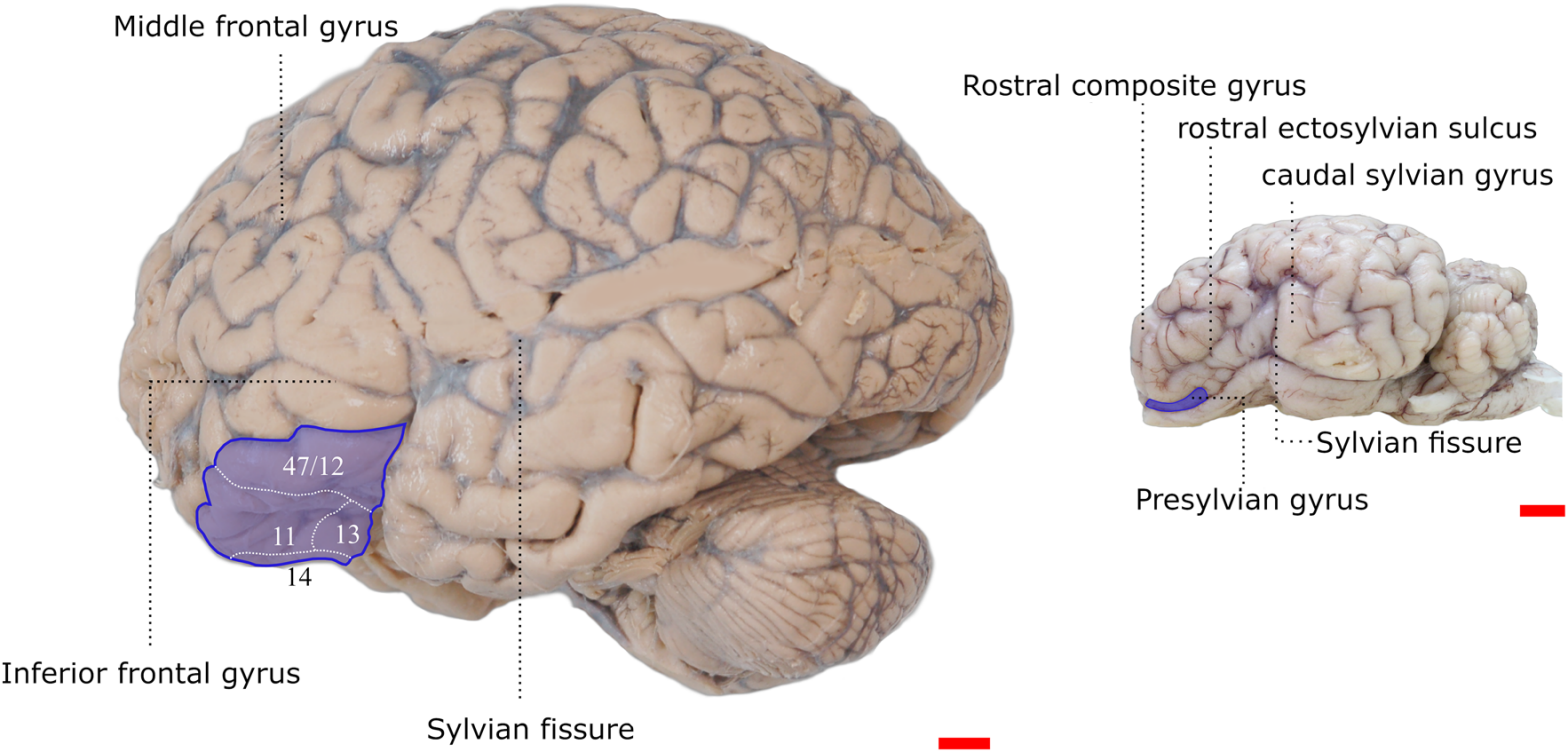
OFC in the human (left) and the sheep (right). In the human, the OFC is divided in Walker’s (1940) and Petrides and Pandya’s (1994) areas. In the sheep, the OFC is the orbital (ventral) part of prorean gyrus. Scalebar = 1 cm

The precise identification of the OFC in mammals other than primates is a matter of debate. In many quadrupedal mammals, including the sheep, the entire PFC matches the prorean gyrus (Rose 1942; Rose and Woolsey 1948), as it receives most of the inputs from the mediodorsal thalamus (Dinopulous et al. 1985; Fuster 2015). The topographical location corresponds to an area ventral to the Presilvian sulcus and is a continuation of the precentral gyrus and rostral composite gyri (Constantinescu 2018; Barone 2004). However, no studies have been performed in the sheep to confirm that the orbital area of the prorean gyrus receives most of the thalamic inputs of the MDmc. A study conducted on 17 Göttingen minipigs showed that the MDmc projects to an area topographically similar to the "orbitofrontal region" of the sheep (Jelsing et al. 2006). A comparison between the two areas considered in this study can be found in Fig. 1.

The neocortex of artiodactyls shows a complex pattern of external gyri, but its cytoarchitecture is rather different from that of primates and rodents and shows a certain reduction of layer IV (Peruffo et al. 2019; Graïc et al. 2021a,b). Consequently, the target of most thalamic inputs must be found elsewhere, potentially in layers I and II (Costantinople and Bruno 2013; Cozzi et al. 2017; La Rosa et al. 2020).

The sheep has acquired a certain interest as a translational species in experimental medicine and surgery (Guillamón and Clau 2010; Jacobsen et al. 2010; Morton and Howland 2013; Sartoretto et al. 2016). Therefore, the study of the OFC in this even-toed ungulate could be of particular interest both as an experimental model and per se. Sheep have been shown to have good memory, usually associated with the prefrontal cortex and seem to be capable to recognize a face and emotions similarly to humans (Kendrick et al. 2001; Knolle et al. 2017). Since the OFC is involved in processing various inputs and regulating emotions, a better understanding of its connections and function in the sheep could also help constitute a foundation for animal welfare studies, considering how little is known of animal production species (Cozzi et al. 2020).

The aim of this paper was to compare the connections and neurochemical properties of the OFC of humans with its topographical equivalent in the sheep. Comparison of connections was based on magnetic resonance images (MRI) and subsequent analyses of the white matter tracts revealed by Diffusion Tensor Imaging (DTI) in the brains of three sheep and five human brains. For analyses of neurochemical properties of the cortical column, we compared the prevalently five-layered sheep cortex with the classical six-layered cortex of the chimpanzee, a nonhuman primate often likened (although obviously not fully equivalent) to humans (Gu and Gu 2003; Gomez-Robles et al. 2016; Graïc et al. 2020). The use of the chimpanzee’s cortices furtherly allowed us to analyze specimens sampled and processed with a comparable timing sequence with those of the sheep, and presumable parallel quality of the outcomes. To this effect we studied the cytoarchitecture of the area and the distribution of the calcium binding proteins (Ca-BPs) Calbindin (CB), Calretinin (CR) and Parvalbumin (PV), using an experimental series composed five sheep and four chimpanzees.

## Materials and methods

### Origin of the specimens

A total of 12 animal brains were analysed (*Ovis aries* *n* = 8, *Pan troglodytes* *n* = 4). DTI scans of human brains (*n* = 5) were obtained from the Human Connectome Project (HCP) database (https://ida.loni.usc.edu/login.jsp). Information can be found in Table 1.

**Table 1:** Data on the origin and nature of the brain and brain samples examined

Species	Internal ID	Sex	Age	Cause of death	Use	Death to extraction interval
*Ovis aries*	Sheep a	F	Adult	Commercial Slaughtering	DTI	10–20’
	Sheep b					
	Sheep c					
	Sheep 1				H / IHC	6 h
	Sheep 2					
	Sheep 3					
	Sheep 4					
	Sheep 5					
*Pan troglodytes*	36,675	M	Adult	Drowning	H / IHC	Within 6 h
	64,361	M		Cardiac arrest		Within 6 h
	68,010	F		Non-CNS related pathologies		Within 6 h
	70,113	M		Heart and respiratory failure		Within 6 h
*Homo sapiens*	MGH 1007	M	Adult	Not applicable	DTI	Not applicable
	MGH 1010	F				
	MGH 1016	M				
	MGH 1019	F				
	MGH 1031	M				

*F* female, *M* male, *DTI* diffusion tensor imaging, *H* histology, *IHC* immunocytochemistry

Sheep samples originate from a commercial slaughterhouse. The sheep were treated according to the European Community Council directive (86/609/EEC) on animal welfare during the commercial slaughtering process and were constantly monitored under mandatory official veterinary medical care. The definition of “adult” was based on the official documentation available corresponding to each individual ear mark and confirmed evaluation of body size by examination of teeth wear. The chimpanzees were brought to the Department of Comparative Biomedicine and Food Science (BCA) of the University of Padova from their outdoor location for post-mortem examination. All animals were previously housed in zoological parks operating under mandatory governmental CITES permits and consequent vigilance. All animals were found dead by the respective wardens. Causes of death were due to heart or system failure unrelated to the nervous system. Post-mortem macro- and microscopic examination of the brain did not identify lesions or anomalies.

The HCP project (Principal Investigators: Bruce Rosen, M.D., Ph.D., Martinos Center at Massachusetts General Hospital; Arthur W. Toga, Ph.D., University of Southern California, Van J. Weeden, M.D., Martinos Center at Massachusetts General Hospital) is supported by the National Institute of Dental and Craniofacial Research (NIDCR), the National Institute of Mental Health (NIMH) and the National Institute of Neurological Disorders and Stroke (NINDS). HCP is the result of efforts of co-investigators from the University of Southern California, Martinos Center for Biomedical Imaging at Massachusetts General Hospital (MGH), Washington University, and the University of Minnesota.

### Tissue sampling

All the animal brains were extracted and fixed in neutral buffered paraformaldehyde (4%). The time interval between the death of the animal and the extraction of the brain are reported in Table 1. All these processes took place at the department.

After at least 1 month of fixation, three sheep brains were transferred to the Department of Neuroscience, Biomedicine and Movement of the University of Verona to perform the MRI using a 4.7 Tesla, 33-cm bore horizontal magnet (Oxford Ltd., Oxford, UK) equipped in a Bruker tomograph (Bruker, Karlsruhe, Germany). The OFCs of the remaining sheep were collected following landmarks available in (Rose 1942; Constantinescu 2018; Barone 2004). Sampling in the chimpanzee brain was performed following directions found in specific atlases (Bailey et al. 1950). In each specimen, we collected an axial section in the middle of the area to include an easily recognizable layer IV.

### Histology and immunocytochemistry

The samples were processed with conventional techniques and then embedded in paraffin for histology and immunocytochemistry studies. For both the processes, samples were then cut into 8 µm sections through a rotatory microtome (LEICA®). The paraffin was removed through three baths of 5 min each and rehydrated through descendent alcohol solutions (100%, 95%, 70%, 50%) and distilled H_2_O for 3 min each.

For histology we used a Nissl protocol. Therefore, sections were immersed in a 0.4% cresyl violet solution for 4 – 20 min depending on the intensity of the staining. Subsequently, they were rinsed in tap water, the result of the staining was rapidly checked under an optic microscope and, when necessary, some sections were immersed in cresyl violet twice or more until they reached a satisfactory result. Finally, they were dehydrated in an increasing alcohol solution series (70%, 95%, 100%) for a few seconds, immersed in xylene (four baths of 3 min each) and cover slipped.

For *immunocytochemistry* sections were immersed in a citrate solution (pH 6) or Tris HCl (Trizma base, pH 9, Sigma-Aldrich®), heated up to 90 °C in a microwave for 10 min and left to cool down at room temperature for 30 – 50 min. They were then washed in three baths of 3 min each of Tris HCl Buffered Saline (TBS). After this, they were moved into a wet chamber and a perimeter with the ImmEdge pen (Vector Laboratories, Inc., Burlingame, CA, USA) was drawn to maintain the volume of the subsequent solutions (150 µL) on the sections and were treated with H_2_O_2_ at 3% for 10 min. After three rinses in TBS, they were incubated 1 h at room temperature then overnight at 4 °C with the primary antibody (Table 2) in a dilution solution (SuMi, 0,5 mL Triton X-100 and 0,25 g of gelatin in 100 mL of TBS). Three rinses in TBS followed and then, the sections were incubated 2 h with the secondary antibody (Table 2) in SuMi or 20 min in HRP (EnVision FLEX system, Dako, USA) solution. After three washes in TBS, samples in HRP were treated with a diaminobenzidine-chromogen Substrate Solution (EnVision Flex) while others were incubated 30 min in Avidine Biotin Complex kit (Vectastain, Vector Labs, Burlingame, UK), rinsed in TBS and incubated in diaminobenzidine-tetrahydrochloride kit (DAB, Merck). Both last incubations lasted 5 – 20 min depending on the intensity of the staining. Consequently, sections were rinsed in distilled H_2_O, counterstained with the Nissl’s protocol described in the previous paragraph and finally cover slipped.

**Table 2:** List of primary and secondary antibodies used for the *immunocytochemistry* process

Primary antibody	Immunogen/host	Supplier	Dilution	Antibody RRID
Calbindin D-28 K	Monoclonal mouse Polyclonal rabbit	Swant, Switzerland	1:1000 1:1000	AB_10000347 AB_10000340
Parvalbumin	Monoclonal mouse		1:1000	AB_10000343
Calretinin	Monoclonal mouse		1:1000	AB_10000320

Secondary antibody	Immunogen/host	Supplier	Dilution	Antibody RRID
Biotinylated anti-mouse	Goat	Vector Labs, Burlingame, CA, USA	1:400	AB_2336171
Biotinylated anti-rabbit	Goat		1:400	AB_2313606

Final slides were scanned with the D-Sight v2 (Menarini Diagnostics srl, Italy), a semi-automated microscope, at 20 X magnification, fast mode and automatic focusing. Images acquired were in Jpeg2000 format and were used, through the program Navi Viewer (Menarini Diagnostics srl, Italy), to calculate the thickness of the cortical layers. These are expressed in mean plus standard deviation and the percentage of the single layer on the total thickness. Three measurements were taken per section, two sections per individual. Those images used for this paper were transformed and elaborated in GIMP (GNU Image Manipulation Program 2.10, www.gimp.org) and Inkscape 1.0 (The Inkscape Project, www.inkscape.org).

### DTI

#### Sheep

Images were obtained with a single coil. The receiver and transmitter was a birdcage with 7.2 cm of inner diameter. A 2D fast acquisition with relaxation enhancement (RARE) series was used to obtain high resolution T2w structural images. The parameters were: RARE factor 16; field of view (FOV) 6.0 × 5.0 cm; repetition time (TR) 35,736 ms; matrix size (MTX) 240 × 200; echo time (TE) 78.1 ms; 160 slices of 0.5 mm in thickness; 0.250 × 0.250 mm resolution; number of averages (NEX) 8. Acquisition time: 1 h and 11 min. DTI images were obtained using an Echo Planar Imaging (EPI) series which settings were: EPI factor 11; FOV 6.0 × 5.0 cm; TR 20,000 ms; MTX 120 × 100; TE 24.7 ms; 80 slices of 1.00 mm; isotropic in-plane resolution of 0.500 mm; NEX 6. In addition, 5 b0 images and 30 noncollinear directions with a b value of 3000 s/mm2 were acquired. Acquisition time: 12 h and 50 min.

The OFC identification and selection were performed with the ITK-SNAP 3.8.0 software (Yushkevich et al. 2006; www.itksnap.org) following the topographic localisation described in the atlases previously named. Then, the regions were uploaded in DSI Studio (www.dsi-studio.labsolver.org) to identify the tracts. Each OFC region was labeled as an "end region" which allowed to build the tracts that started or arrived from/to the region.

#### Humans

**Fig. 2:**
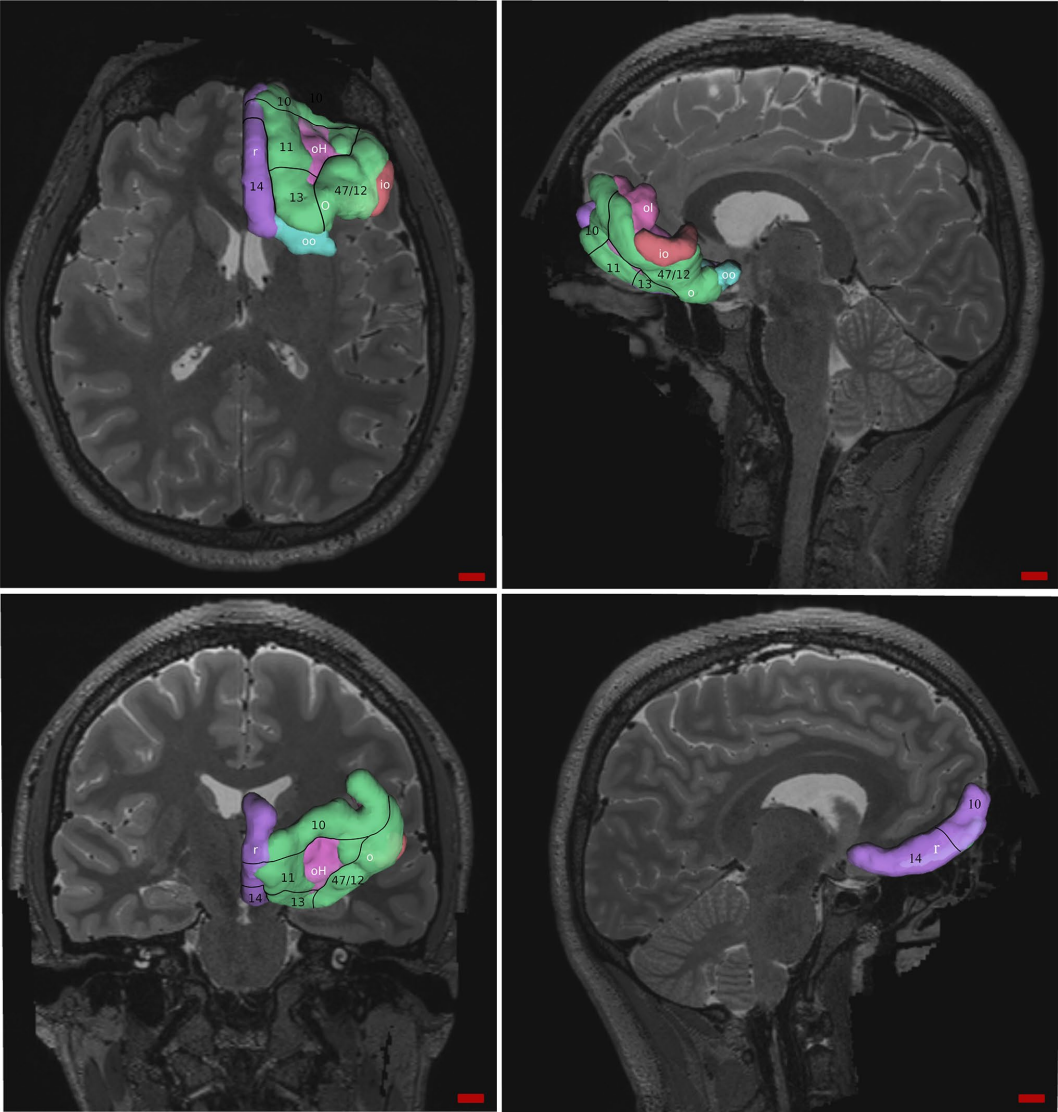
OFC representation in human following the standardized parcellation. Walker’s and Petrides and Pandya’s areas are written in black and delimited by black lines. White letters indicate the template segments: io, “G_front_inf-Orbital”; o, “G_orbital”; r, “G_rectus”; ol, “S_orital_lateral”; oo, “S_orbital_med-olfact” and oH, “S_orbital-H_shaped”

Details about data acquisition and processing can be found at the following link: www.humanconnectome.org/study/hcp-young-adult/document/mgh-adult-diffusion-data-acquisition-details/. In this case, we used the templates provided in DSI Studio (options “Atlas” > ”FreeSurferSeg”) based on the standardized nomenclature given by Destrieux et al. (2010). We chose the segments “G_front_inf-Orbital”, “G_orbital”, “G_rectus”, “S_orital_lateral”, “S_orbital_med-olfact” and “S_orbital-H_shaped”, which corresponded to “Orbital part of the inferior frontal gyrus”, “Orbital gyri”, “Gyrus rectus”, “Lateral orbital sulcus”, “Olfactory sulcus” and “Orbital sulci”, respectively. In these parcellations the areas 11, 47/12, 13 and 14 were present, although some of them also occupied area 10 (Fig. 2). The tracts of the former were deleted.

In both species, we set the fractional anisotropy (FA) threshold at 0.1 and the angle threshold at 45°.

## Results

In the current study, we did not find major differences between the brains of the same species. Sheep OFCs were topographically and microscopically similar and consistent among specimens and the connections shown by tractography were analogous in location but not in quantity. Chimpanzee OFCs had also uniform characteristics and no main changes were found, as for other areas in previous works (Cozzi et al. 2017; Graïc et al. 2020). The tracts from human brains reflected nearly the same pattern among individuals.

In each section, the sheep will be first described, as it was the object of this research, and then compared to the chimpanzee.

### Histology with Nissl stain

**Fig. 3:**
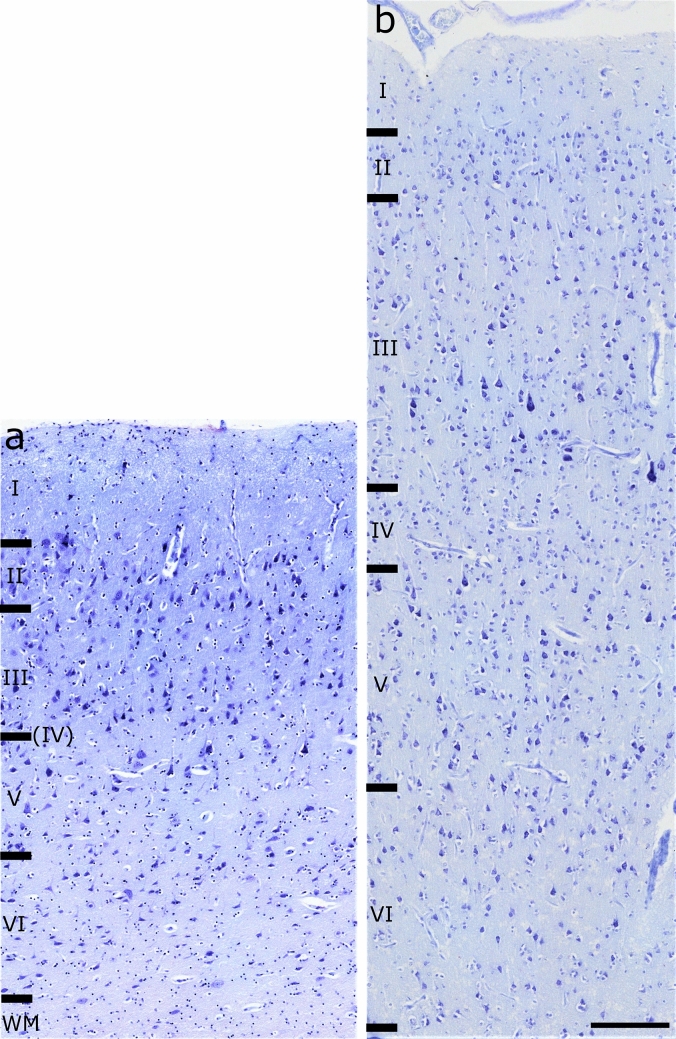
OFC layering in the (**a**) sheep and (**b**) the chimpanzee. WM, white matter. Nissl stain. Scale bar 200 µm

In the sheep, layer IV was absent in all the sampled tissue (Fig. 3a) and we did not observe any rostro-caudal development. Layers I, III, V and VI were relatively equally distributed (range 20.2 – 26.2%) except for layer II (range 9.2 – 9.9%).

In the chimpanzee, on the other hand, a layer IV was present (Fig. 3b) and we found percentages of similar thickness for layers III, V and VI, whereas layer II was reduced by 40% and layer I more than doubled (Table 3).

**Table 3:** Thickness of the total cortical column and single layers of the species analyzed

	Sheep			Chimpanzee		
	Mean	SD	%	Mean	SD	%
Total	1696.54	164.54		2479.46	142.25	
I	356.67	48.52	21	215.53	13.80	8.7
II	167.16	39.38	9.9	141.87	10.75	5.7
III	380.95	64.47	22.5	651.13	58.93	26.3
IV	–	–	–	149.06	34.99	6
V	347.55	64.91	20.5	559.75	79.02	22.6
VI	443.03	91.46	26.1	761.75	83.74	30.7

Values of mean and SD are expressed in µm

*SD* standard deviation

### Immunocytochemistry for Ca-BPs

**Fig. 4:**
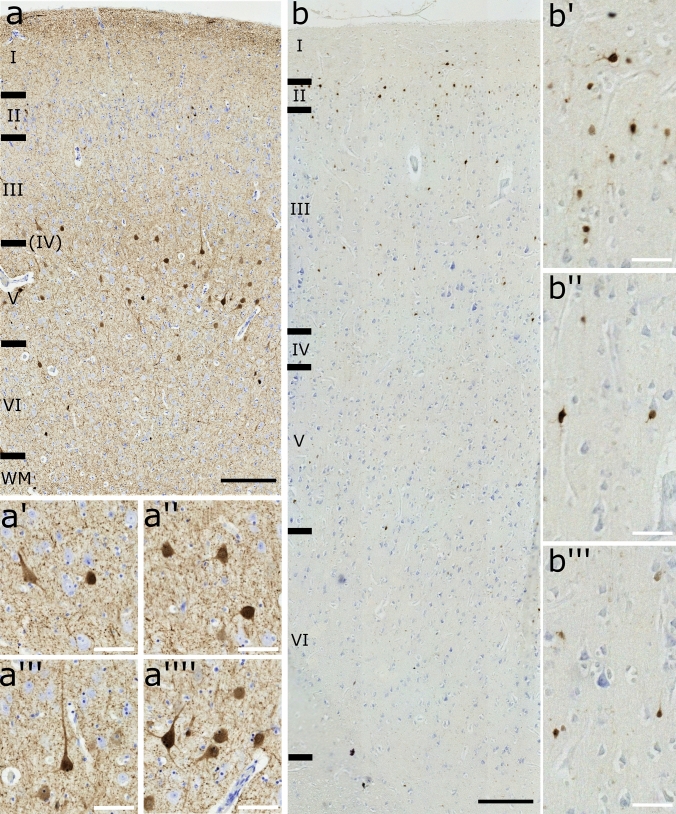
Transversal cut of the OFC. PV**-ir** neurons in (**a**) sheep and (**b**) chimpanzee. **a’**, interneurons in layer II; **a’’**, two interneurons in layer III; **a’’’**, a pyramidal cell close to an interneuron in layer V; **a’’’’**, pyramidal neuron in layer V. **b’**, interneurons in layer III; **b’’**, interneuron in layer V; **b’’’**, interneuron in layer III. Black scale bar 200 µm; red scale bar 50 µm

**Fig. 5:**
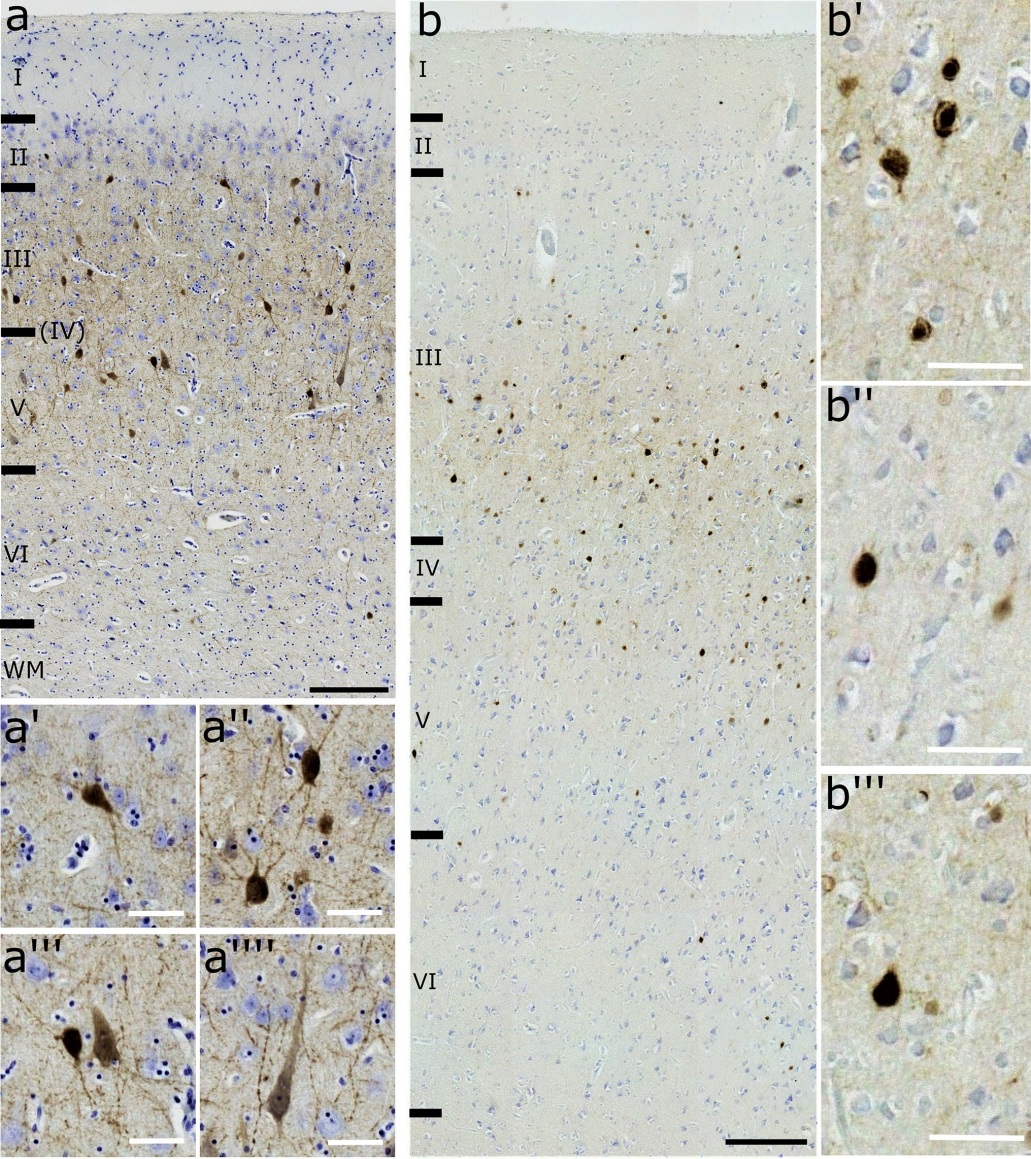
Transversal cut of the OFC. CR-ir neurons (**a**) sheep and (**b**) chimpanzee. **a′**, presumptive pyramidal neuron and an interneuron in layer III; **a′′**, interneurons between layers III and V; **a′′′**, pyramidal cell close to an interneuron in layer V; **a′′′′**, pyramidal neuron with a group of interneurons in layer V. **b′**, interneurons in layers I and II; **b′′**, interneurons in layer III; **b′′′**, pyramidal cell in layer III. Black scale bar 200 µm; red scale bar 50 µm

**Fig. 6:**
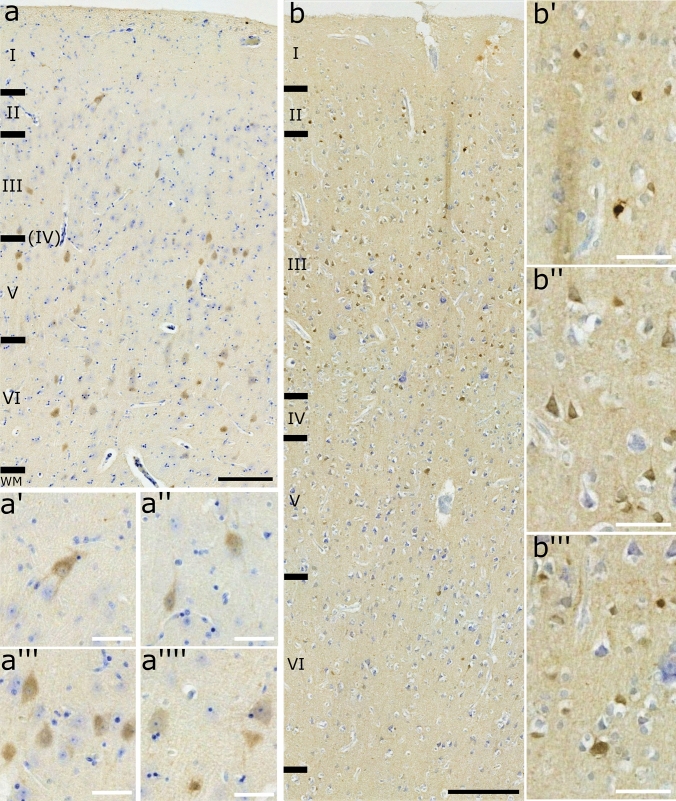
Transversal cut of the OFC. CB-ir neurons (**a**) sheep and (**b**) chimpanzee. **a’** and **a’’**, interneurons in layer II and III respectively;** a′′′**, group of interneurons in layer V; **a′′′′**, two presumptive pyramidal neurons in layer VI.**b′** and **b′′′**, interneurons in layers II/III and IV respectively; **b′′**, pyramidal neurons in layer III. Black scale bar 200 µm; red scale bar 50 µm

Staining with Ca-BPs revealed immunoreactive (-ir) neurons in the examined species for all the three antibodies (Figs. 4, 5 and 6). Their distribution spanned throughout the cortex with some differences.

In the sheep, PV-ir neurons were mostly present in layers III and V and fewer were generally found in layer II (Fig. 4a). Most of them were interneurons with various shapes (Fig. 4a’ and 4a’’) but few pyramidal neurons were also found, mostly in layer V (Fig. 4a’’’ and 4a’’’’). The chimpanzees showed a distribution of PV-ir neurons more clustered in layer III, followed by layers V, VI, II and IV (Fig. 4b). Only interneurons were marked (Figs. 4b’ – 4b’’’).

In the sheep, CR-ir neurons were mainly present in layer V and to a lesser extent in layers III and VI (Fig. 5a). They were mostly interneurons (Fig. 5a′ – a′′′′); however, pyramidal neurons were also found both in layer III (Fig. 5a’) and V (5a′′′, 5a′′′′). In the chimpanzee, CR-ir neurons were seemingly more present in layer II and less in layers I, III, V and VI (Fig. 5b). They were mostly interneurons (Figs. 5b′, 5b′′) but also pyramidal neurons were found (Fig. 5b′′′).

In the sheep, CB-ir neurons were evenly distributed along the cortex, with relatively more neurons found in layer VI (Fig. 6a). Many of them were interneurons in layers II (Fig. 6a′), III (Fig. 6a′′), V (Fig. 6a”’) and VI. However, pyramidal neurons were mostly found in layer VI (Fig. 6a’’’’). In the chimpanzee, CB-ir neurons were found mainly in layer III but could also be seen in other layers (Fig. 6b). We observed interneurons in layers II (Fig. 6b′), layer III (Fig. 6b′), layer IV (6b′′′) and layer V (Fig. 6b). Pyramidal neurons were present only in layer III (Fig. 6b′′).

### DTI

**Fig. 7:**
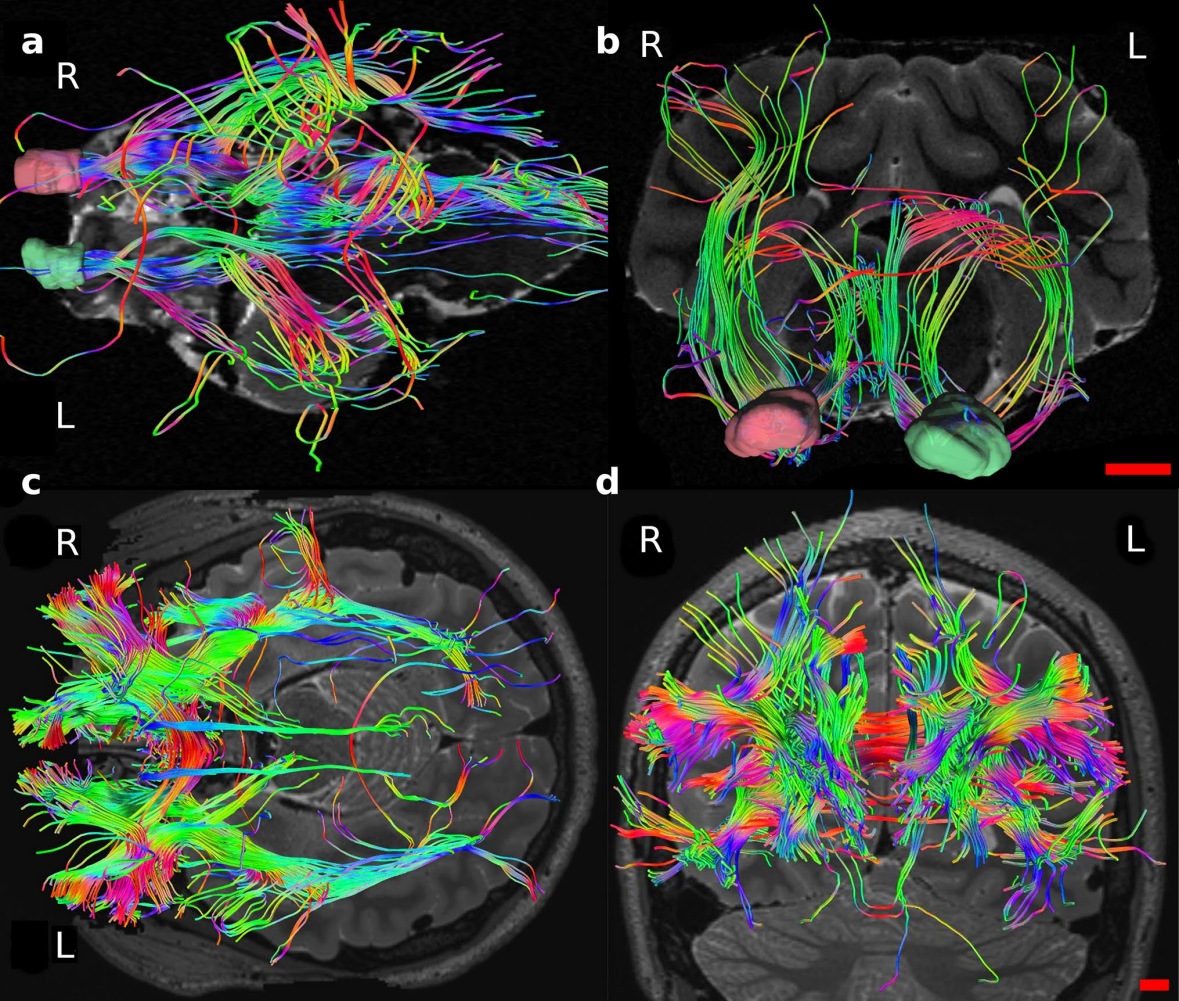
Tractography of the sheep (top row) and human (bottom row) related to the OFC. **a** and **c** are axial views, **b** and **d** are coronal views

A whole tractographic image showing tracts from the OFC is in Fig. 7.

#### Sheep

**Fig. 8:**
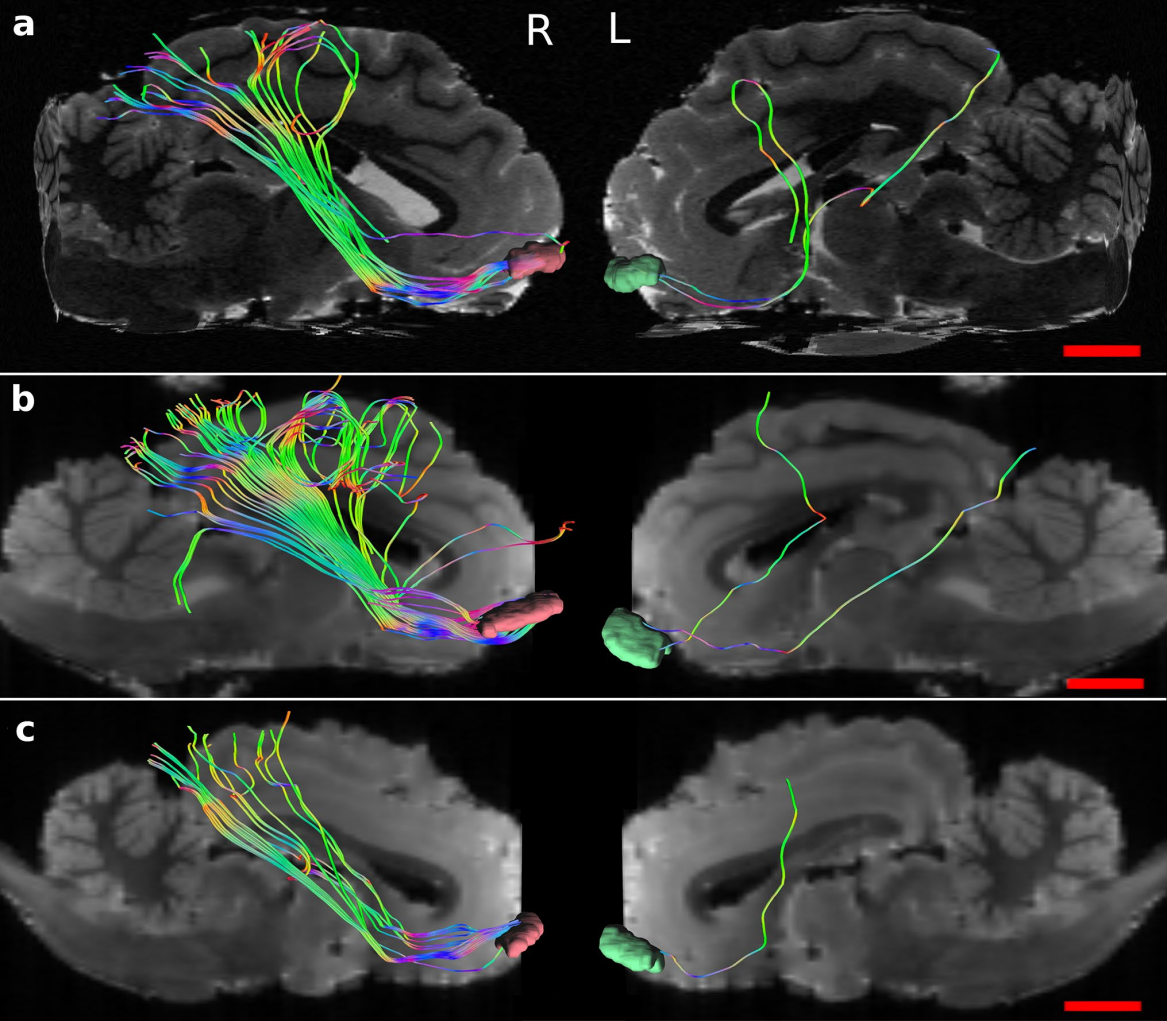
Sagittal views of the three sheep specimens (**a**, **b** and **c**) in the right (R) and left (L) sides. Note the difference in cortico-cortical fibers between the two hemispheres, reaching the visual and auditory primary areas on the right side. These tracts were specifically isolated from the others

**Fig. 9:**
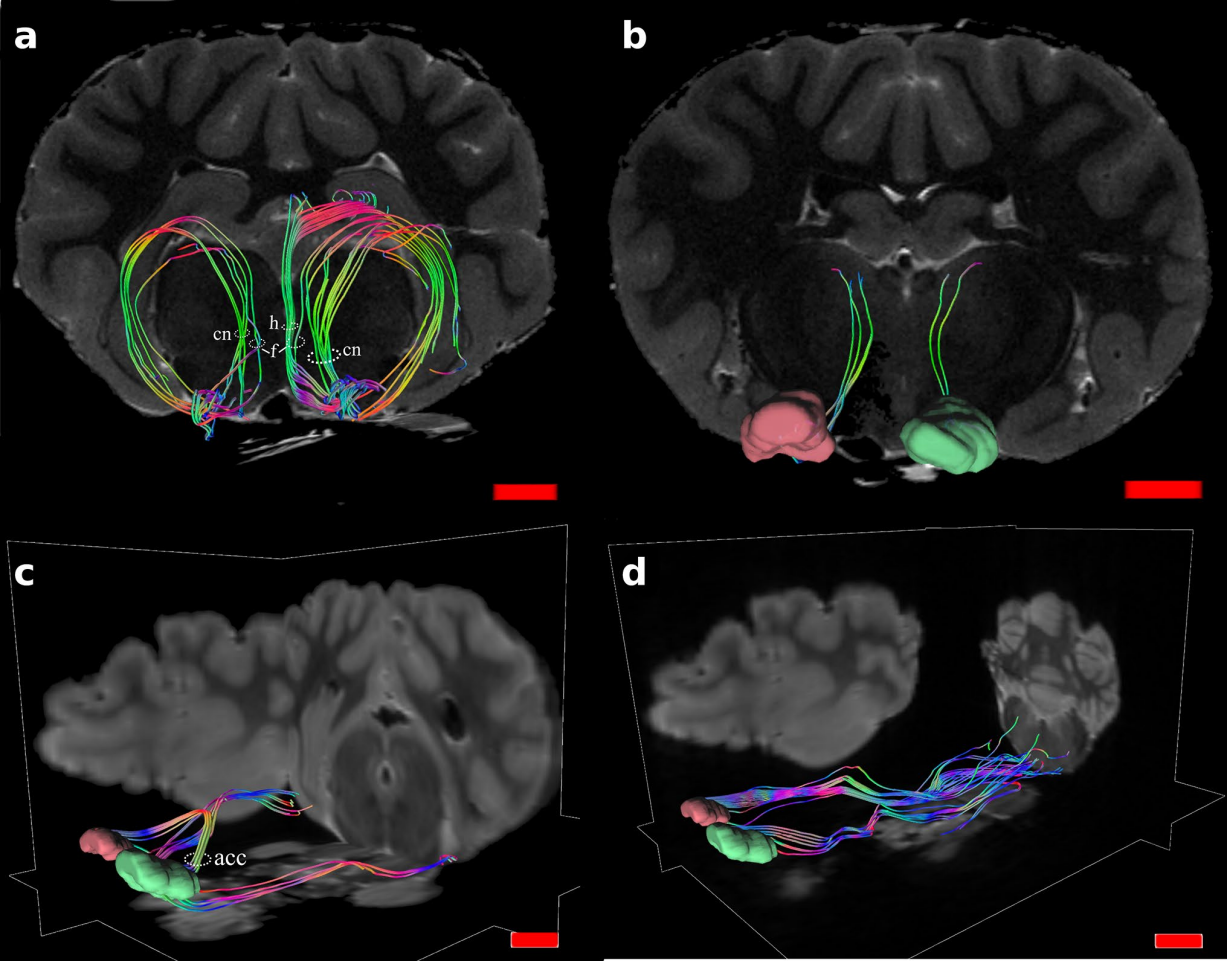
**a** Coronal view of sheep connections related with caudate nucleus (cn), fornix (f) and hippocampus (h); **b** coronal view showing links with the thalamus MD nucleus; oblique views of **c**) connections related with piriform cortex, claustrum and participation to the anterior corpus callosum (acc) and **d** corticofugal tracts

Tractography showed connections between the OFC and several areas. Most important and relevant were ipsilateral connections with the visual areas following the inferior fronto-occipital fasciculus (ifof) in which we consistently found a marked asymmetry between the two hemispheres. Very few fibers running to acoustic areas were also present (Fig. 8). Connections included areas related to the caudate nucleus, the fornix and the hippocampus (Fig. 9a), the nucleus medialis dorsalis of the thalamus (MD) (Fig. 9b), the piriform cortex and the claustrum (Fig. 9b). Finally, other connections were reciprocal through the anterior corpus callosum (acc, Fig. 9b) and corticofugal: they passed through to the ventral striatum, lateral hypothalamus, divided in periaqueductal gray, red nucleus and substantia nigra to then reach the spinal cord (Fig. 9c).

#### Human

**Fig. 10:**
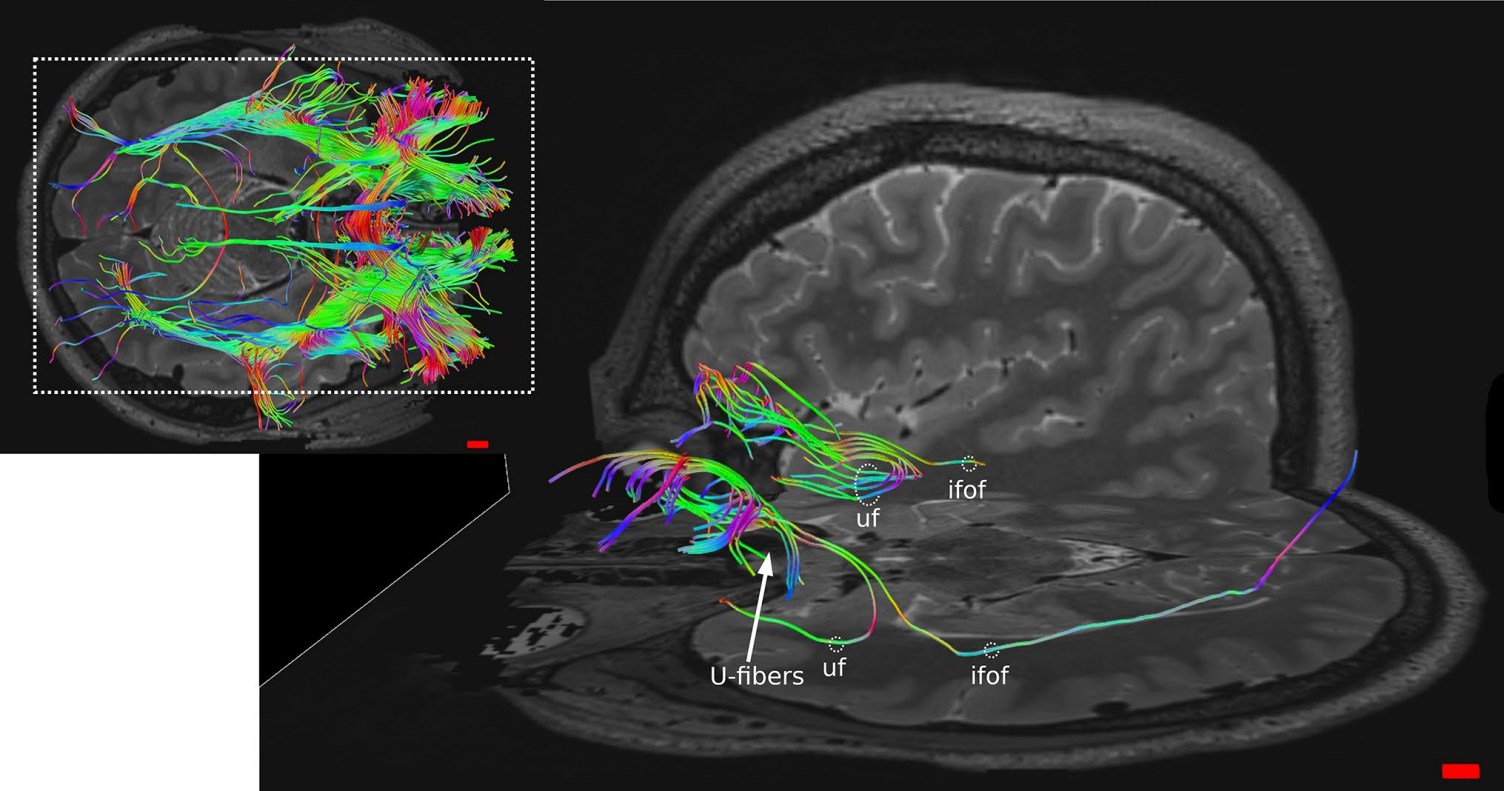
Whole OFC tractography in human in axial view (top left); magnification of Area 11 connections. ifof, inferior fronto-occipital fasciculus; uf, uncinate fasciculus

Area 11 was connected to areas 10 and 47/12 and its fibers participated to the uncinate fasciculus (uf) and ifof, going to visual areas. We also found “U fibers” within this area (Fig. 10).

**Fig. 11:**
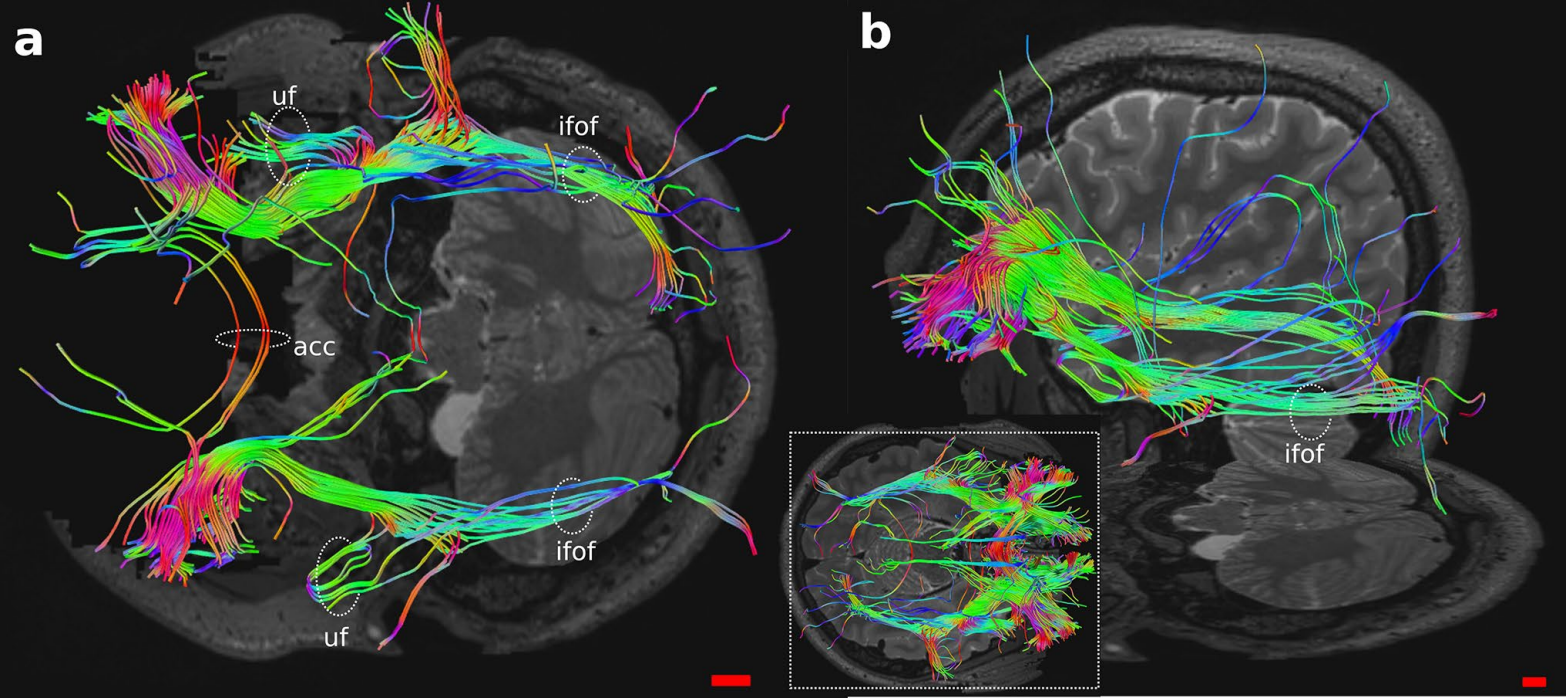
Whole OFC tractography in human (bottom middle); magnification in **a** axial and **b** oblique views of Area 47/12 connections. acc, anterior corpus callosum; ifof, inferior fronto-occipital fasciculus; uf, uncinate fasciculus

Area 47/12 fibers mostly participated to the ifof, going primarily to visual areas and then auditory and gustative areas. Its fibers also constituted the uf and the acc. Other connections were with areas 9, 10, 11, 9/46, amygdala, insular cortex and the thalamus. Even in this area “U fibers” within it were found (Fig. 11).

**Fig. 12:**
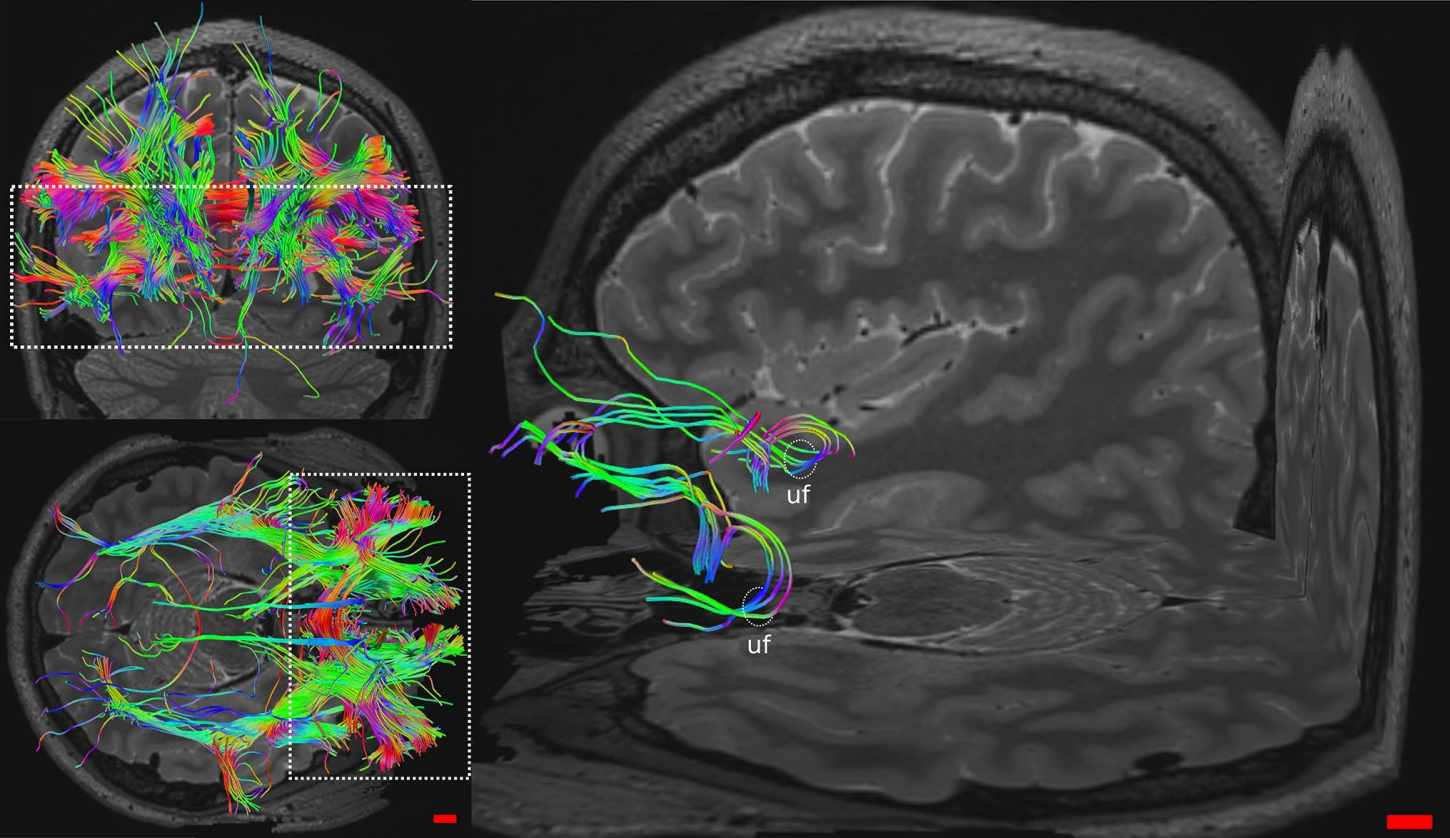
Whole OFC tractography in human in coronal and axial views (left); magnification in oblique view of Area 13 connections; uf, uncinat fasciculus

Area 13 participated mostly to the uf and was connected also to areas 10, 11, 47/12. Its fibers passed through the amygdala and the insular cortex (Fig. 12).

**Fig. 13:**
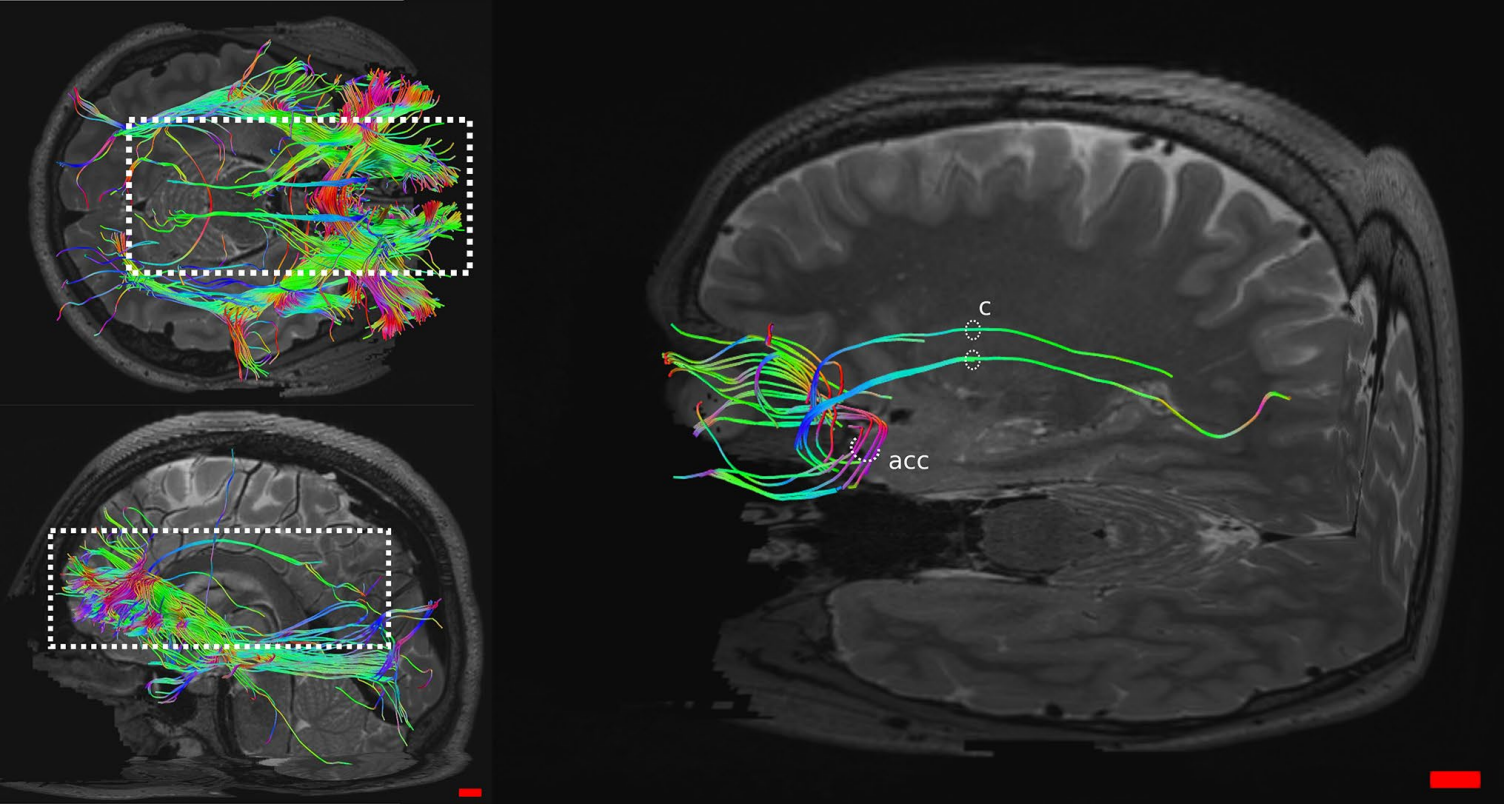
Whole OFC tractography in human in axial and sagittal views (left); magnification in oblique view of Area 14 connections; c, cingulum

Area 14 shown fibers to cingulate cortex, acc and areas 10 and 11 (Fig. 13).

## Discussion

In this study we compared the OFC of the sheep with the human corresponding areas using tractography, and the cytoarchitecture and neurochemistry of the OFC cortical columns with those of the chimpanzee. The human OFC is deemed to be the location of higher brain functions (Tranel et al. 2003) and the connections associated with the topographical analogue area in the sheep could suggest similar functions. Cortical tissues of the chimpanzees are no equivalent substitute for the human, but the organization of its six-layered column of a great ape may represent an acceptable model for a comparison with the sheep. Furthermore, the cortical columns of the sheep and chimpanzees employed in the current study underwent similar processing phases and manipulations.

DTI is a non-invasive technique which is used to show the connectivity of the brain white matter (Jeurissen et al. 2019). Although the FA and other parameters might change in *ex-vivo* tissues, several works have shown this technique gives valuable results on fixed brains, as long as these have been perfused *in-vivo* or otherwise fixed through immersion within a few hours after death. In addition, a fixed brain would hypothetically allow an unlimited acquisition time, increased *b* value, increased number of directions, thus achieving what cannot be done in living tissue (D’Arceuil and de Crespigny 2007; Rane and Duong, 2011; Wang et al. 2018). Historically, Golgi staining and anterograde and retrograde tracing were the first procedures used to study the connections in the nervous system (Rose and Woosley 1948; Dinopoulos et al. 1985). However, DTI is more advantageously applicable in larger brains, where tracing is more difficult to perform and extremely time consuming. Inherently limited, the current anatomical value of DTI has been extensively discussed (Schilling et al. 2020) and caution is warranted in the interpretation of the tracts.

### Cytoarchitecture and connections

As expected, Nissl staining revealed the complete absence of layer IV in the sheep over the entire length of the region, whereas this layer was found in the chimpanzee (Fig. 3). Notably, layer I in sheep was more developed in proportion compared to the chimpanzee (21% *vs.* 8.7%) (Table 3). This is consistent with the literature and could be consistent with a higher reliance on layer I for cortico-cortical connections. Other studies confirmed the absence of layer IV in *Artiodactyla* (Hof et al. 1999) and, specifically in the sheep, the “*area orbitalis*” of Rose (1942) lacked the same layer as well. The OFC in the chimpanzees was indeed sampled to find the layer IV, due to its rostro-caudal development (Petrides and Pandya 2012).

The immunocytochemistry in both species showed higher concentration of PV-ir neurons in layer III and V. Higher concentration of CR-ir neurons were found in layer V in the sheep and layer II in the chimpanzee. Finally, a uniform distribution of CB-ir neurons along the cortex was evident for both species. As observed by Hof and collegues (1999, 2000) and Hof and Sherwood (2005), CaBP-ir neurons had a similar distribution between rats and primates but both differed from artiodactyls. Studies in rats and primates reported that CB-ir and CR-ir neurons were mostly distributed in layers II and III even though other groups present in the other layers were identified. In both cases, they were interneurons and weakly stained pyramidal neurons. On the other hand, PV-ir neurons were found more frequently in layers III-V and another small group in layers II and III (Hof et al. 1999, 2000; Hof and Sherwood 2005). Some pyramidal neurons were also marked in few functional areas in some but not all species (DeFelipe et al. 1997). This was similarly consistent with our results. Indeed, CB-ir and CR-ir were mainly interneurons located primarily in layer III followed by layers I, II and V. PV-ir neurons, present in layer III then V, VI were only interneurons. Furthermore, the organization and neurochemical organization of the orbitofrontal cortex of the chimpanzee has previously been reported in the literature to be largely similar to the human OFC (Petrides and Pandya 1994; Öngür and Price 2000).

Literature concerning artiodactyls described that, while CB-ir neurons were weakly stained and fewer compared to CR-ir neurons, both of them were large multipolar, bipolar or fusiform cells in layers I, II, and superficial III, and a few in layers V and VI. Furthermore, other ungulates such as camels, llamas and giraffes showed more CR-ir neurons than the sheep. In dolphins, in layer IIIc/V some neurons were weakly stained CR-ir and CB-ir pyramidal cells. On the other hand, PV-ir interneurons were limitedly distributed (5%) related to CR-ir and CB-ir cells (40%). In whales and dolphins, these marked interneurons were located in layers IIIc/V, whilst few pyramidal neurons also in layer III (Glezer et al. 1993; Hof et al. 1999, 2000; Hof and Sherwood 2005). In the present study, while our data corresponds to the literature for CB (Fig. 6), we did not find CR-ir cells in all layers, as we did for CB (Fig. 5). Additionally, PV-ir cells were clearly distributed mostly along layer III/V but pyramidal cells were found mostly in layer V rather than III (Fig. 4). PV-ir pyramidal neurons likely play an inhibitory neuromodulatory role in layer V, although studies in other areas indicate the possibility of an excitatory function at least partially (Meskenaite, 1997; Liu et al. 2014). The greater concentration of CR-ir neurons in layer V, compared to all other layers in the literature, could indicate a modified inhibitory function at that level.

The lack of layer IV and the diverse distribution of CaBPs might imply a different integrative architecture in the minicolumn of the OFC and potentially more broadly another organization of neuronal circuits in sheep compared to humans.

The DTI revealed both similarities and differences between human and sheep white matter tracts. Indeed, while in the human brain the areas corresponding to the OFC are four, in the sheep only one region identified based on current models (Ella et al. 2017). Notably, some tracts were remarkably analogous to human albeit some other tracts were "novel" in the sheep. In the human brain, area 47/12 had connections with more areas than areas 11, 13 and 14 (Fig. 11). Its fibers participated mostly to the uf, ifof, which linked it to the visual and acoustic areas and then the acc. Other connections were to the thalamus, other prefrontal areas such as 9, 10, 11, 9/46 and within the same area (U-shaped). Area 11 contributed to uf, ifof, reached to areas 10 and 47/12 and presented U-shaped fibers (Fig. 10). Area 13 had intra prefrontal connections with areas 10, 11 and 47/12 and joined in the uf (Fig. 12). Finally, area 14 was related to area 10, 11 and its streams to acc and cingulate cortex (Fig. 13).

General agreement on the OFC connections derived from studies, using tracing and audiography, mostly in rhesus monkey (*Macaca mulatta*), are resumed by reviews of Kringelbach and Rolls (2004) and Petrides and Pandya (2012) (see Table 4 below).

**Table 4:** Scheme of the connections concerning the OFC’s single areas or the OFC as a whole

OFC’s area	Connected areas and bundle presence	Reference
11	9/**46v**, **10**, **47/12**, **13**, **14**, **23**, **24**, **28**, **30**, **32**, **35**, **45**, 46, **insula**, **OPro**, *posterior hypothalamus*, **rostral TC**, **SII**, superior temporal sulcus, **TPro**, **ventromedial temporal lobe**	*Kringelbach and Rolls*, (2004)*;* **Petrides and Pandya, **(2012)
47/12	**6**, **9**, 9/**46v**, **10**, **11**, **13**, **24**, **32**, **44**, **45**, *Am*, dysgranular insula, *inferotemporal cortex*, *posterior hypothalamus*, **ProM**, SII, TPro	*Kringelbach and Rolls*, (2004)*;* **Petrides and Pandya, **(2012)
13	**10**, **11**, **47/12**, **14**, **45**, **46**, *Am*, insula, *entorhinal*, perirhinal and gustatory areas, **OPro**, *PC*, **ProM**, **temporal lobe rostral and ventro-medial parts**	*Kringelbach and Rolls*, (2004)*;* **Petrides** **and** **Pandya,** (2012)
14	9, **10**, **11**, **47/12**, **13**, **24**, **25**, **28**, **32**, **35**, **45**, 46, *Am*, *H*, **OPro**, **TPro**	*Kringelbach and Rolls*, (2004)*;* **Petrides** **and** **Pandya** (2012)
OFC as whole	*CN, preoptic region, VTA, periacqueductal gray,* claustrum bundles: ifof, uf acc	*Kringelbach and Rolls*, (2004)*;* Fernàndez-Miranda et al. (2008); Petrides and Pandya, (2012) Kier et al. (2004); Hau et al. (2017); Conner et al. (2018); Baynes et al. ( 2002)

Italic or bold words mean that the information is present only in one of the bibliographic sources, whereas no italic or bold words means that the information is present in all the sources

*acc* anterior corpus callosum, *Am* amygdala, *CN* caudate nucleus, *H* hippocampus, *ifof* inferior fronto-occipital fasciculus, *OPro* orbital proisocortex, *SII* secondary somatosensory area, *PC* piriform cortex, *ProM* motor proisocortex, *TC* temporal cortex, *TPro* temporal proisocortex, *uf* uncinate fasciculus, *VTA* ventral tegmental area

Based on the data files acquired from the HCP, we compared our results with the literature. The analyzed DTI lacked specific tracts and connections to some regions, but we did not find any “extra” tract previously undescribed.

In the sheep, the associations we found were with the caudate nucleus, fornix/hippocampus, claustrum, piriform cortex and acc. Interestingly, we found a large amount of corticofugal fibers and a marked right asymmetry of cortico-cortical fibers (ifof) which connected the OFC primarily to visual areas (Figs. 8 and 9).

In the sheep, tracing related to this area has been performed but data are scarce. The first study dates back to 1948 when Rose and Woolsey attempted to demonstrate whether an area homologous to the primate OFC existed in the cat, the sheep and the rabbit. In their work, they considered that frontal fields receive projections from the mediodorsal nucleus of the thalamus. However, the authors did not distinguish the various components of the mediodorsal nucleus of the thalamus, with their respective projections, and considered the OFC to be the entire PFC. Retrograde tracing was performed in just one sheep, but the results showed a much larger lesion area than the OFC they had previously found (Rose 1942). Most likely the tracer had extended slightly excessively; however, the general results should not be discarded. A subsequent study was carried out by Dinopoulos and colleagues (1985) who inoculated, on the contrary, horseradish peroxidase into various areas of the rostral prorean gyrus in ten sheep. It resulted in lesions not only to the MD nucleus of the thalamus but also to the mediodorsal part of the ventromedial nucleus, and to a lesser extent to the posterior lateral nucleus and the midline nuclei. The authors concluded that the prefrontal cortex in sheep corresponded to the OFC of Rose and Woolsey (1948) and the "*regio frontalis*" of Rose (1942), although they did not analyze the ventral aspect of the gyrus. These studies focused only on the connections with the nuclei of the thalamus and ignored connections with other areas of the brain. A recent research by Meurisse and colleagues (2008) demonstrated the existence of a relationship between the cortical nuclei of the amygdala with the OFC in sheep, pointing out its implication in olfactory stimuli elaboration. Despite the paucity of data on the OFC, we trust that our results are coherent with the characteristics of the OFC. Indeed, some fibers connected to the thalamus MD and certain similarities in the rest of the connections with that of the human indicate a certain robustness of the method and results.

From our tractography results, the lack of the aforementioned tracts in both species could be related to software or acquisition biases. In the sheep, the large amount of corticofugal fibers could be caused by an artifact. In effect, fibers reaching the hypothalamus as an input/output was not unexpected (Rempel-Clower and barbas 1998; Kringelbach and Rolls 2004); however, their continuation to the spinal cord in a unique bundle is quite doubtful. The hypothalamus is an integration center of visceral/autonomic mono- and polysynaptic inputs (Cameron 2001; Saper 2012). It is possible that, at this level, these fibers aligned with other ones and, having the voxels the same anisotropy, these were mixed by the algorithm and resulted as a single stream.

To summarize, while there are extensive data on OFC in primates, more concerning tracing than DTI, little is known about sheep and other artiodactyls. Given the lack of existing studies on neurofiber tracing in sheep, we could not establish accurate correspondence between the two species. However, our digital results and homologies established from literature data in this animal suggested some degree of similarity compared to the human brain. Further tracing studies could be carried out, dividing parts of the MD nucleus of the thalamus to establish precise areas.

### Functional considerations

The OFC is considered to be the cortical representation of the limbic system (Mtui et al. 2020). It is involved in reward-punishment evaluation after primary and secondary reinforcements (Hof et al. 1995; Kringelbach and Rolls 2004; Rudebeck and Rich 2018). A leucotomy of the OFC in fact produced uninhibited and aggressive behavior, hence it was originally thought to have an inhibitory role on emotions (Kringelbach and Rolls 2004; Mtui et al. 2020; Rudebeck and Rich 2018). Other types of lesions showed that the OFC was more important for decision making rather than inhibition. This has been explained by the fact that leucotomies most probably also affect areas close to the OFC thereby causing more extensive reactions while excitotoxic lesions were more targeted. Currently, most authors accept the involvement of the OFC in both functions (Kringelbach and Rolls 2004; Rudebeck and Rich 2018).

To date, no frontal leucotomy has been performed in the sheep, therefore we cannot assume or predict what effect such an experiment would have on the specific behavior in this species. However, there have been several recent non-invasive surveys using functional near infrared spectroscopy (fNIRS) to study brain emotions and functions in large mammal brains including sheep (Min et al. 2012; Gygax et al. 2013; Vögeli et al. 2014, 2015; Guldimann et al. 2015; Gygax and Vögeli 2016; Chincarini et al. 2020). Most of them did not point out the possibility that the fNIRS probe could not reach the frontal fields given the shape of the skull in the species used. While in the study of Vögeli and colleagues (2014) the sensor was too rostral to reach orbital cortices, Chincarini and collegues’ (2020) results were related to the motor areas instead of the prefrontal ones due to this anatomical feature. Nonetheless, based on our findings, the presence of connections with the fornix/hippocampus, claustrum, piriform cortex, hypothalamus, visual areas and the absence of U-shaped fibers, cingulum and fibers with other parts of the prorean gyrus (remaining part of the prefrontal cortex), we can infer that in the sheep the area responsible for reward and punishment elaboration and emotional control, the “orbitofrontal cortex” in primates, putatively partially share the same function.

So far, brain lateralization has been well established in humans as well as in some other species (Güntürkün et al. 2020). Although relatively weak, there seems to exist a certain lateralization of function within the orbitofrontal cortex (Lopez-Persem et al. 2020). Actually, reward and positive emotions seem to be more represented on the left hemisphere, whilst the punishment and negative emotions in the right hemisphere (O’Doherty et al. 2001; Alves et al. 2008; Leliveld et al. 2013; Mtui et al. 2020; Güntürkün et al. 2020).

Similarly, in the sheep, a differentiation between the right and left brain hemisphere has been demonstrated through behavioral studies. In fact, it is generally accepted that sheep face recognition, with its possible negative behavioral and emotional consequences (Da Costa et al. 2004; Knolle et al. 2017), are predominant in the right hemisphere (Peirce et al. 2000, 2001; Peirce and Kendrick 2002; Da Costa et al. 2004; Versace et al. 2007). Although from DTI we could not determine quantitatively the cortico-cortical fibers reaching the visual areas were conveying signal in or out from the OFC, the strong relationship between the two areas suggested a high specialization and the importance of one hemisphere’s function over the other. Being phylogenetically a prey, the sheep has an existential incentive to recognize other members of its flock from a dangerous situation, such as the presence of a wolf or another predator. The elaboration of this assessment might have brought this species to lateralize this function to the right hemisphere.

## Data Availability

Data used for this manuscript are either publicly available (Human Connectome Project (Principal Investigators: Bruce Rosen, M.D., Ph.D., Arthur W. Toga, Ph.D., Van J. Weeden, M.D.). HCP funding was provided by the National Institute of Dental and Craniofacial Research (NIDCR), the National Institute of Mental Health (NIMH) and the National Institute of Neurological Disorders and Stroke (NINDS). HCP data are disseminated by the Laboratory of Neuro Imaging at the University of Southern California) or are available from the corresponding author on reasonable request.

## Abbreviations

acc: Anterior corpus callosus
BCA: Department of comparative biomedicine and food science
Ca-BPs: Calcium binding proteins
CB: Calbindin
CR: Calretinin
DTI: Diffusion tensor imaging
fNIRS: Functional near infrared spectroscopy
HCP: Human connectome project
ifof: Inferior fronto-occipital fasciculus
ir-: Immunoreactive
MD: Nucleus medialis dorsalis of the thalamus
MDmc: Medial (magnocellular) nucleus of the mediodorsal thalamus
MGH: Massachusetts general hospital
NIDCR: National institute of dental and craniofacial research
NIMH: National institute of mental health
NINDs: National institute of neurological disorders and stroke
OFC: Orbitofrontal cortex
PFC: Prefrontal cortex
ProM: Motor proisocortex
PV: Parvalbumin
SII: Secondary somatosensory area
uf: Uncinate fasciculus
